# Three new species of the genus *Falcileptoneta* Komatsu, 1970 (Araneae, Leptonetidae) from Korea

**DOI:** 10.3897/zookeys.872.34594

**Published:** 2019-08-20

**Authors:** Mingjie Xu, Seung Tae Kim, Jung Sun Yoo, Eun Jung Nam, Shuqiang Li

**Affiliations:** 1 Hebei Key Laboratory of Animal Diversity, College of Life Science, Langfang Normal University, Langfang 065000, China Institute of Zoology, Chinese Academy of Sciences Beijing China; 2 Institute of Zoology, Chinese Academy of Sciences, Beijing 100101, China Langfang Normal University Langfang China; 3 Life and Environment Research Institute, Konkuk University, Seoul 05029, Korea Konkuk University Seoul South Korea; 4 Division of Animal Resources, National Institute of Biological Resources, Incheon 22689, Korea Division of Animal Resources, National Institute of Biological Resources Incheon South Korea

**Keywords:** Diagnosis, morphology, new species, taxonomy

## Abstract

Three new species of the genus *Falcileptoneta* Komatsu, 1970 belonging to the spider family Leptonetidae Simon 1890 are described from Korea. All species were collected from wet leaf litter layers.

## Introduction

The family Leptonetidae Simon, 1890 currently contains 21 genera and 349 species worldwide (World Spider Catalog 2019). Most species are tiny (1–3 mm) and have six eyes with the posterior median eyes located behind the posterior lateral eyes; some species have only four or two eyes or are eyeless. Most species live in irregular sheet webs in leaf litter, caves, or mines. In Korea, there are 42 species in four genera: *Leptoneta* Simon, 1872, *Falcileptoneta* Komatsu, 1970, *Masirana* Kishida, 1942 and *Longileptoneta* Seo, 2015. Komatsu (1970) erected *Falcileptoneta* with *F. striatus* (Oi 1952) as the type species (transferred from *Leptoneta*). Prior to this study, 47 *Falcileptoneta* species have been described from Korea and Honshu, Shikoku in Japan (Irie and Ono 2007). In this study, three new species of *Falcileptoneta* are illustrated and described.

## Materials and methods

All specimens were collected by hand from mountainous districts in northern South Korea. Type specimens are deposited in the National Institute of Biological Resources (**NIBR**) in Incheon, Korea and the Institute of Zoology, Chinese Academy of Sciences (**IZCAS**) in Beijing, China. All spiders were preserved in 95% ethanol and examined under a LEICA M205C stereomicroscope. Images were captured with an Olympus C7070 wide zoom digital camera (7.1 megapixels) mounted on an Olympus SZX12 dissecting microscope, and Helicon Focus image stacking software was used to compile the images. All images were edited with Adobe Photoshop CS8.1. Methods follow those of Wang and Li (2011) and Ledford et al. (2011); terminology follows Wang et al (2017). The map was drawn with the assistance of ArcGIS 10.2 and edited with Adobe Photoshop CS8.1. All measurements are in millimeters (mm). The left male palps are illustrated. Leg measurements are displayed as total length (femur, patella, tibia, metatarsus, tarsus). Leg segments were measured on their dorsal side. Abbreviations of morphological structures are as follows:

**At** atrium;

**E** embolus;

**MS** median sclerite;

**SS** spermathecae stalk;

**PS** prolateral sclerite;

**SH** spermathecae.

## Taxonomy

### Family Leptonetidae Simon, 1890

#### Genus Falcileptoneta

Taxon classificationAnimaliaAraneaeLeptonetidae

Komatsu, 1970

037C77793F8D5440AC0E6B857F2C39BA

##### Type species.

*Leptoneta striatus* Oi, 1952

##### Diagnosis.

The genus *Falcileptoneta* is similar to *Leptoneta* and *Longileptoneta* by having fewer sclerites on the bulb but can be distinguished from these two genera by the combination of the following characters: tarsus of male palp with shallow, transverse depression and without spines; tibia with apophyses on the retrolateral apical end; bulb with sickle-like or membranous embolus; complex laminae of the bulb. *Leptoneta* can be distinguished by the tarsus with a branch bearing an apical spine. *Longileptoneta* can be identified by the strong femoral spines, the prolateral curvature bearing a prolateral distal spur on the tarsus, a finger-like median sclerite, and a leaf-like embolus.

##### Comments.

According to the original description, the genus *Falcileptoneta* is similar to *Leptoneta* but can be distinguished by the falcula on the tibia of the male palp. Femur normal. Tibia with three trichobothria, and the apical end with spine-like apophyses. Tarsus with several bristles but no spines and a weak transverse depression. We have added descriptions of the following characters: the shape of the embolus and the laminae of the bulb.

##### Distribution.

Korea, Japan.

#### Falcileptoneta baegunsanensis sp. nov.

Taxon classificationAnimaliaAraneaeLeptonetidae

B8A28E5B08F45C579F1F89C0BC4BEC4C

http://zoobank.org/3DAFCF04-0F6A-43D9-B945-6369F2126A10

Figures 1 , 2 , 7

##### Type material.

***Holotype:*** male (NIBR), Mt. Baegunsan, Seonyudam Valley near the parking area (38.0702°N, 127.4026°E), Dopyeong-ri, Idong-myeon, Pocheon-si, Gyeonggi-do, Korea, 09 October 2018, ZG. Chen, Z. Zhao & MJ. Xu leg. ***Paratypes***: 1 male and 1 female (IZCAS), same data as holotype.

##### Etymology.

The specific name is an adjective referring to the type locality.

##### Differential diagnosis.

**Figure 1.**
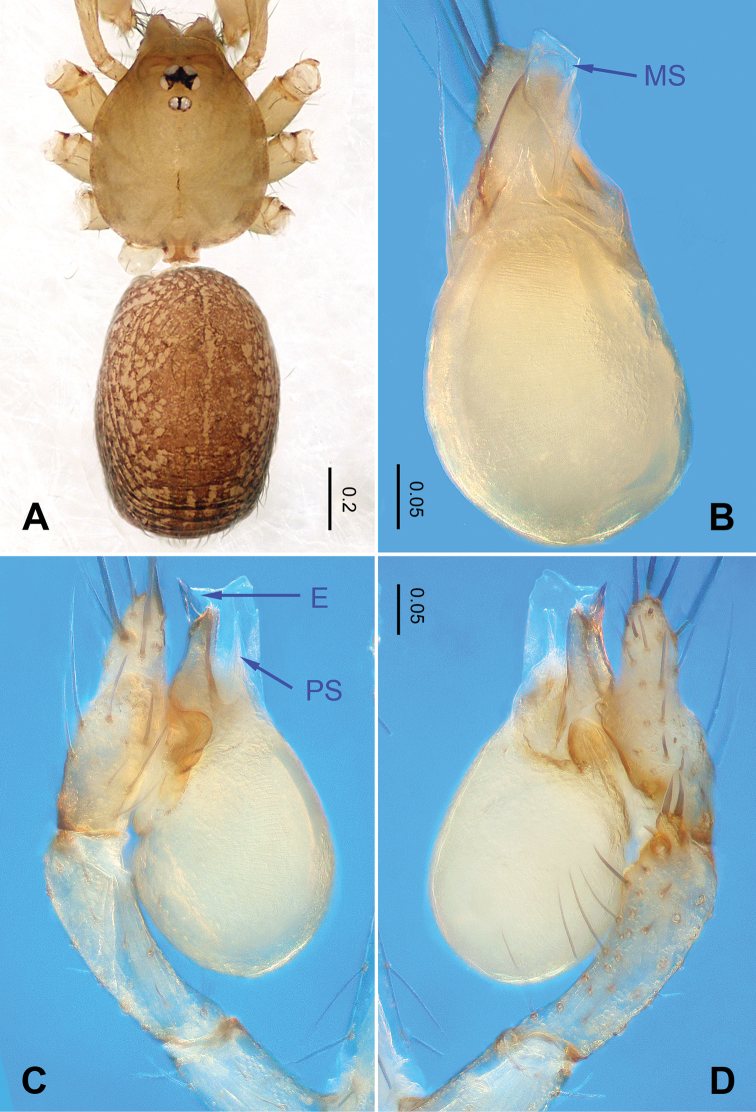
*Falcileptoneta baegunsanensis* sp. nov., male holotype. **A** habitus, dorsal view **B** palpal bulb, ventral view **C** palp, prolateral view **D** palp, retrolateral view. Abbreviations: **PS** = prolateral sclerite; **E** = embolus; **MS** = median sclerite.

This new species is similar to *F. geumsanensis* Seo, 2016 and *F. boeunensis* Seo, 2015 but can be separated by the shape of the palpal tibial apophyses found retrolaterally, with one apophysis coniform and the other bifurcate, bearing three spines distally (Figure 1D) (vs. a spur-like tibial apophysis located ventrally, a leaf-like medial apophysis, a dorsal beak-like apophysis in *F. geumsanensis*; three tibial spines with the outer spine spur-like, the medial one spine-like, and the inner spine conical in *F. boeunensis*.).

##### Description.

***Male*** (holotype). Total length 1.40. Prosoma 0.60 long, 0.51 wide. Opisthosoma 0.80 long, 0.55 wide (Figure 1A). Prosoma brown. Eyes six. Median groove, cervical grooves and radial furrows distinct. Clypeus 0.15 high. Opisthosoma gray, ovoid. Leg measurements: I 3.53 (0.94, 0.25,0.99, 0.74, 0.61); II 2.97 (0.88, 0.23, 0.74, 0.62, 0.50); III 2.58 (0.66, 0.19, 0.68, 0.63, 0.42); IV 3.4 (0.98, 0.19, 1.00, 0.81, 0.42). Palp as illustrated in Figure 1C, D: femur lacking strong spine; tibia with three apophyses retrolaterally, with one coniform and the other bifurcate, bearing three spines distally (Figure 1D); tarsus with a transverse depression, and a smooth, distinct earlobe-shaped process. Bulb with triangular black sickle-shaped embolus and three types of sclerites: prolateral sclerite membranous; median sclerite wide and shoehorn-like; retrolateral sclerite transparent and membranous (Figure 1B).

**Figure 2.**
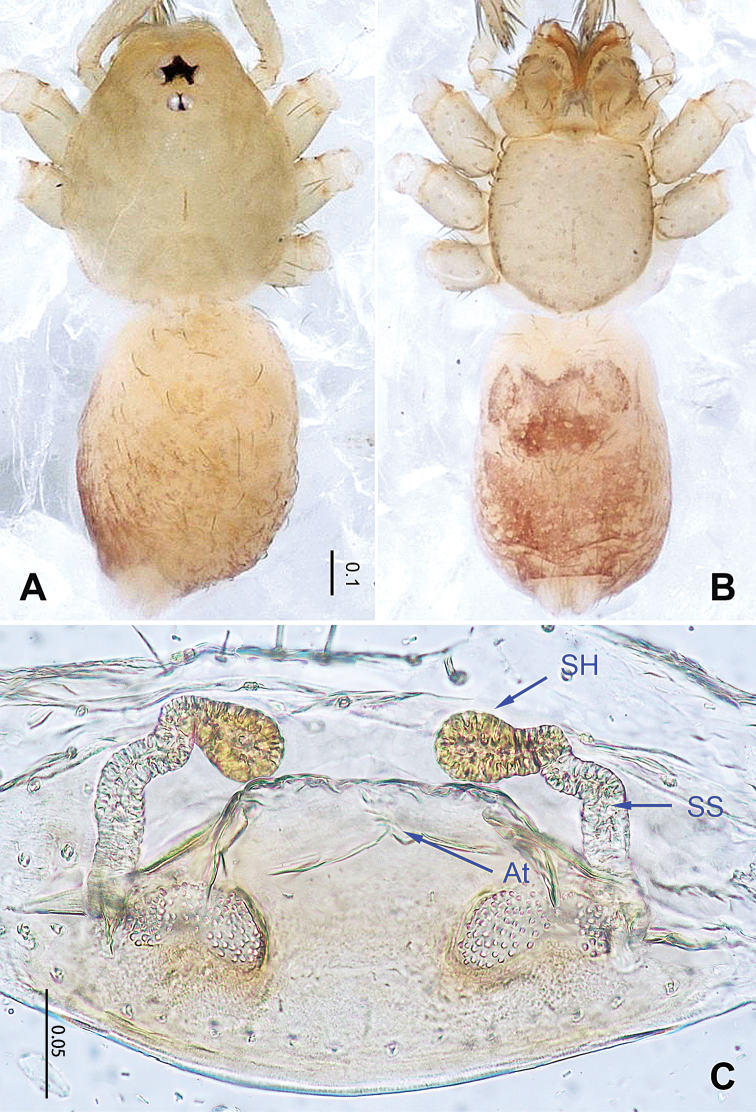
*Falcileptoneta baegunsanensis* sp. nov., female paratype. **A** habitus, dorsal view **B** habitus, ventral view **C** internal genitalia, dorsal view. Abbreviations: **At** = atrium; **SS** = spermathecae stalk; **SH** = spermathecae.

***Female*** (one of the paratypes). Similar to male in color and general features but larger and with longer legs. Total length 1.39 as in Figure 2A, B. Prosoma 0.55 long, 0.46 wide. Opisthosoma 0.84 long, 0.50 wide. Leg measurements: I 3.25 (0.78, 0.18, 0.84, 0.98, 0.47); II 2.29 (0.69, 0.17, 0.60, 0.48, 0.35); III 2.09 (0.56, 0.16, 0.52, 0.45, 0.40); IV2.88 (0.84, 0.22, 0.80, 0.63, 0.39). Internal genitalia as provided in Figure 2C: atrium rectangular, anterior margin of atrium with short hairs, and spermathecae oval.

##### Habitat.

Litter layers in mixed forest.

##### Distribution.

Korea (Gyeonggi-do).

#### Falcileptoneta odaesanensis sp. nov.

Taxon classificationAnimaliaAraneaeLeptonetidae

977DBE5C95715EEF85D704A2499D6DAB

http://zoobank.org/B861FF33-505A-492F-8D88-35C6BB04D47B

Figures 3 , 4 , 7

##### Type material.

***Holotype:*** male (NIBR), Mt. Odaesan 0.2 km east of Sangwonsa Temple (37.7865°N, 128.5665°E), Jinbu-myeon, Pyeongchang-gun, Gangwon-do, Korea, 18 September 2018, ZG. Chen, Z. Zhao & MJ. Xu leg. ***Paratypes***: 1 male and 1 female (IZCAS), same data as holotype.

##### Etymology.

The specific name is an adjective referring to the type locality.

##### Differential diagnosis.

**Figure 3.**
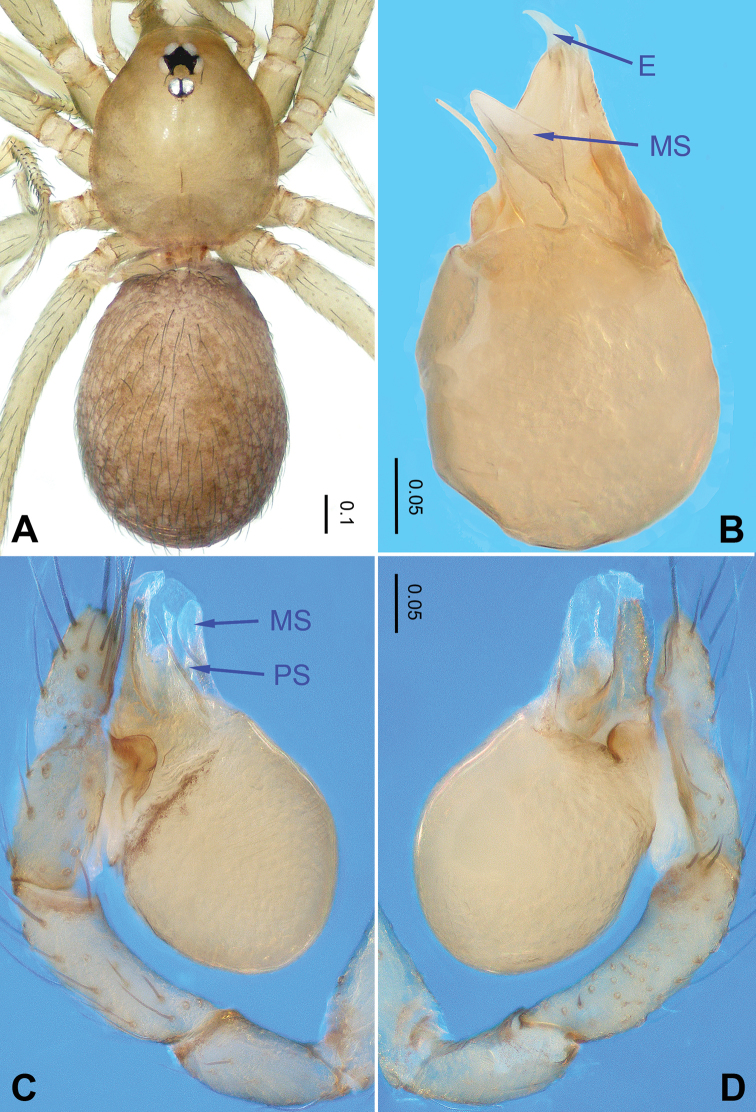
*Falcileptoneta odaesanensis* sp. nov., male holotype. **A** habitus, dorsal view **B** palpal bulb, ventral view **C** palp, prolateral view **D** palp, retrolateral view. Abbreviations: **PS** = prolateral sclerite; **E** = embolus; **MS** = median sclerite.

This new species is similar to *F. geumdaensis* Seo, 2016, *F. sunchangensis* Seo, 2016 and *F. amakusaensis* Irie & Ono, 2005 but can be separated by the palpal tibia with three distal retrolateral setae (Figure 3D) (vs. tibial apophysis with two whip-shaped hairs bearing minute setae and the main apophysis spiniform in *F. amakusaensis*; tibial apophysis with two spur-like retrolateral apophyses in *F. geumdaensis*; tibial apophysis with two retrolateral spines with the dorsal one thick and spur-like in *F. sunchangensis*).

##### Description.

***Male*** (holotype). Total length 1.44 (Figure 3A). Carapace 0.63 long, 0.59 wide. Opisthosoma 0.81 long, 0.59 wide. Prosoma brown. Eyes six, reduced to white vestiges. Median groove, distinct cervical grooves and radial furrows. Opisthosoma gray, ovoid. Leg measurements: I 3.27 (0.80, 0.20, 0.91, 0.70, 0.66); II 2.84 (0.75, 0.21, 0.78, 0.60, 0.50); III 2.36 (0.60, 0.18, 0.55, 0.58, 0.45); IV missing. Male palp as in Figure 3C, D: femur without strong spine; tibia with three retrolateral setae distally, arranged in a triangle; tarsus with a transverse depression (Figure 3D). Embolus with a membranous margin and three types of sclerites: prolateral sclerite spine-like; median sclerite shoehorn-like; retrolateral sclerite membranous (Figure 3B).

**Figure 4.**
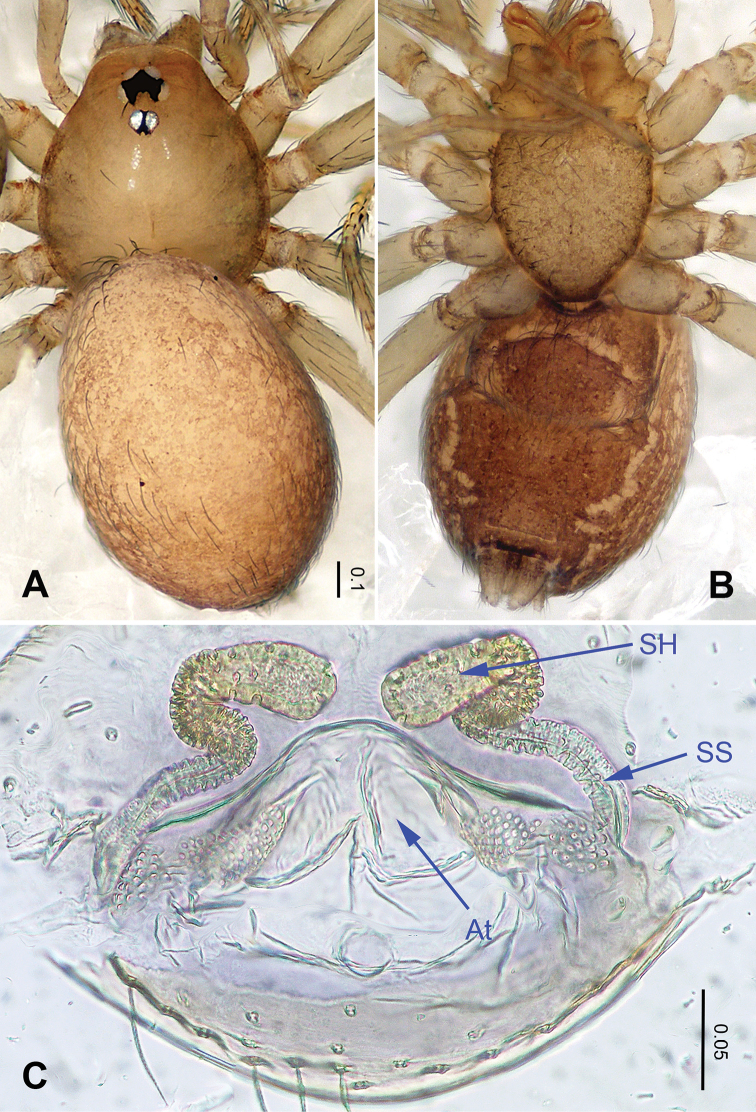
*Falcileptoneta odaesanensis* sp. nov., female paratype. **A** habitus, dorsal view **B** habitus, ventral view **C** internal genitalia, dorsal view. Abbreviations: **At** = atrium; **SS** = spermathecae stalk; **SH** = spermathecae.

***Female*** (one of the paratypes). Similar to male in color and general features but larger and with longer legs. Total length 1.61 as in Figure 4A, B. Prosoma 0.63 long, 0.58 wide. Opisthosoma 0.98 long, 0.69 wide. Leg measurements: I 3.27 (0.80, 0.20, 0.91, 0.70, 0.66); II 2.84 (0.75, 0.21, 0.78, 0.60, 0.50); III 2.36 (0.60, 0.18, 0.55, 0.58, 0.45); IV missing. Internal genitalia as in Figure 4C: atrium triangular, genital duct coiled apically, and spermathecae oval.

##### Habitat.

Litter layers in mixed forest.

##### Distribution.

Korea (Gangwon-do).

#### Falcileptoneta umyeonsanensis sp. nov.

Taxon classificationAnimaliaAraneaeLeptonetidae

8A8D83BBFB465B778840A72317A1BAB6

http://zoobank.org/77593E93-102C-4E98-A900-AD3270FF86C0

Figures 5 , 6 , 7

##### Type material.

**Holotype**: male (NIBR), Mt. Umyeonsan, Seocho-gu, Seoul, Korea, (37.4794°N, 127.0315°E), 03 October 2018, ZG. Chen, Z. Zhao & MJ. Xu leg. **Paratypes**: 1 male and 1 female (IZCAS), same data as holotype.

##### Etymology.

The specific name is an adjective referring to the type locality.

##### Differential diagnosis.

**Figure 5.**
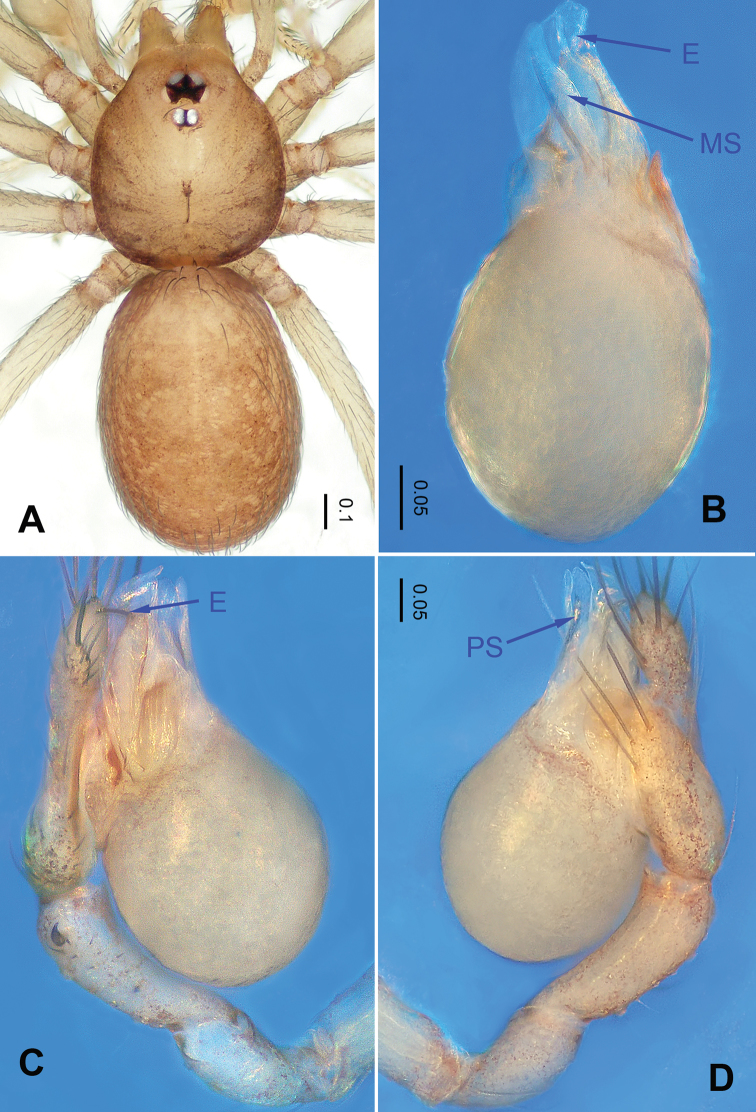
*Falcileptoneta umyeonsanensis* sp. nov., male holotype. **A** habitus, dorsal view **B** right palpal bulb, ventral view **C** right palp, retrolateral view **D** right palp, prolateral view. Abbreviations: **PS** = prolateral sclerite; **E** = embolus; **MS** = median sclerite.

This new species is similar to *F. yebongsanensis* Kim, Lee & Namkung, 2004 and *Leptoneta kwangreungensis* Kim, Jung, Kim & Lee, 2004 but can be separated by one hook-like retrolateral apophysis on the palpal tibia, leaf-shaped median apophysis, and sickle-shaped embolus (Figure 5C) (vs. short tubercle without spines or bristles on tibia, hook-shaped median apophysis and sickle-shaped embolus in *F. yebongsanensis*; spine-shaped embolus and median apophysis in *L. kwangreungensis*).

##### Description.

***Male*** (holotype). Total length 1.58 (Figure 5A). Carapace 0.64 long, 0.57 wide. Opisthosoma 0.94 long, 0.67 wide. Prosoma brown. Eyes six. Median groove, distinct cervical grooves and radial furrows. Opisthosoma brown, ovoid. Leg measurements: I 3.95 (1.09, 0.19, 1.19, 0.84, 0.64); II 3.06 (0.86, 0.18, 0.86, 0.64, 0.52); III 2.51 (0.71, 0.16, 0.67, 0.59, 0.38); IV 4.28 (1.12, 0.21, 1.29, 0.94, 0.72). Male palp as in Figure 5C, D: femur without a strong spine; tibia with one hook-like retrolateral apophysis (Figure 5C). Embolus with a sickle-shaped tip and three types of sclerites: prolateral sclerite spine-like; median sclerite leaf-like; retrolateral sclerite membranous (Figure 5B).

**Figure 6.**
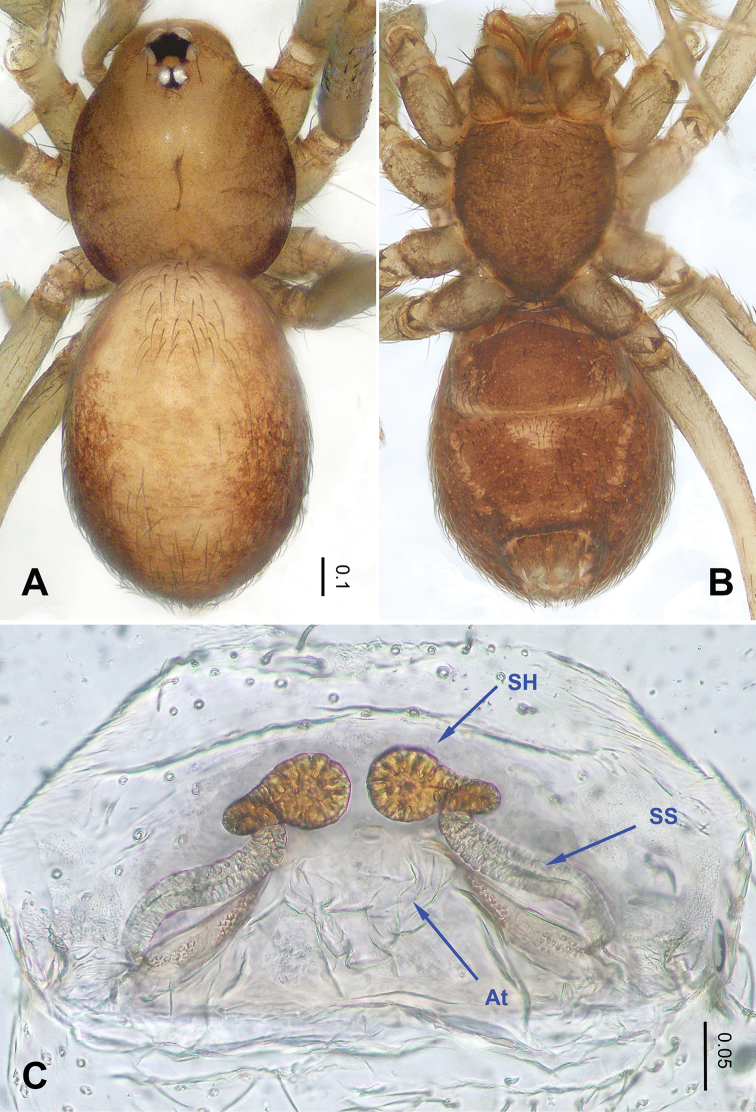
*Falcileptoneta umyeonsanensis* sp. nov., female paratype. **A** habitus, dorsal view **B** habitus, ventral view **C** internal genitalia, dorsal view. Abbreviations: **At** = atrium; **SS** = spermathecae stalk; **SH** = spermathecae.

***Female*** (one of the paratypes). Similar to male in color and general features. Total length 1.51 (Figure 6A, B). Prosoma 0.63 long, 0.55 wide. Opisthosoma 0.88 long, 0.87 wide. Leg measurements: I 3.50 (0.92, 0.20, 0.98, 0.78, 0.62); II 2.84 (0.75, 0.20, 0.76, 0.63, 0.50); III 2.61 (0.69, 0.20, 0.63, 0.61, 0.48); IV 3.71 (0.94, 0.22, 1.00, 0.80, 0.75). Internal genitalia as in Figure 6C: atrium wrinkled, trapezoidal, genital duct coiled apically, and spermathecae round.

##### Habitat.

Litter layers in mixed forest.

##### Distribution.

Korea (Seoul).

**Figure 7.**
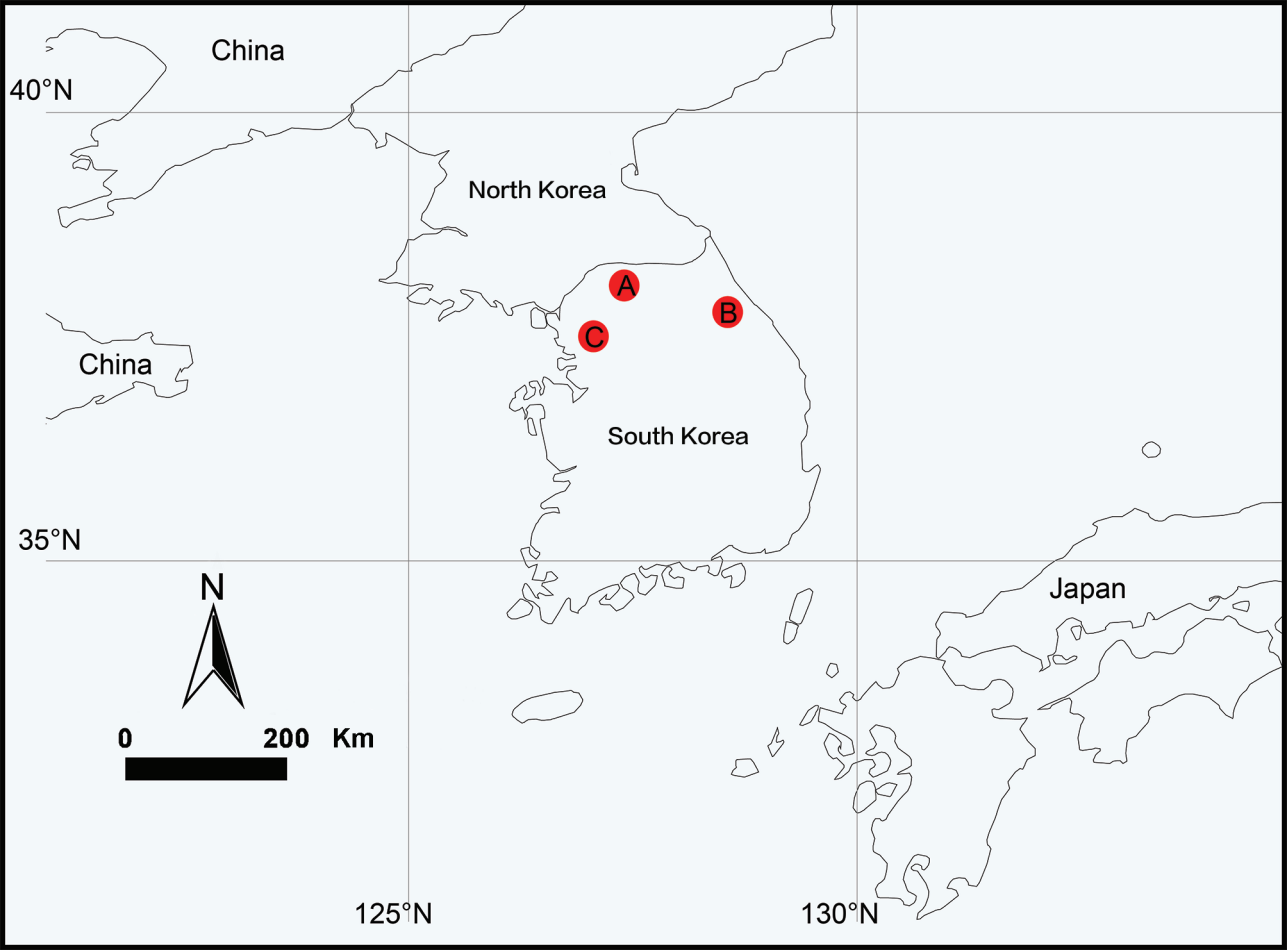
Locality records for three new species of the genus *Falcileptoneta* from Korea. **A***F. baegunsanensis* sp. nov. **B***F. odaesanensis* sp. nov. **C***F. umyeonsanensis* sp. nov.

## Supplementary Material

XML Treatment for Falcileptoneta

XML Treatment for Falcileptoneta baegunsanensis

XML Treatment for Falcileptoneta odaesanensis

XML Treatment for Falcileptoneta umyeonsanensis

## References

[B1] Irie T Ono H (2007) Two new species of the genus *Falcileptoneta* (Arachnida, Araneae, Leptonetidae) collected from Chûbu District, Honshu. Bulletin of the National Museum of Nature and Science, Tokyo, Series A 33: 175–180.

[B2] Irie T Ono H (2005) Seven new species of the genera *Falcileptoneta* and *Masirana* (Araneae, Leptonetidae) from Kyushu. Bulletin of the National Museum of Nature and Science, Tokyo, Series A 31: 77–92.

[B3] Komatsu T (1970) A new genus and a new species of Japanese spiders (*Falcileptoneta* n. g. and *Sarutana kawasawai* n. sp., Leptonetidae). Acta Arachnologica 23: 1–12. 10.2476/asjaa.23.1

[B4] Kim ST Lee JH Namkung J (2004) Two new ground-inhabiting *Leptoneta* spiders (Araneae: Leptonetidae) from Korea. Journal of Asia-Pacific Entomology 7: 257–261. 10.1016/S1226-8615(08)60225-3

[B5] Kim ST Jung MP Kim HS Lee JH (2004) Two new species of litter-inhabiting spiders of the genus *Leptoneta* from Korea (Araneae: Leptonetidae). The Canadian Entomologist 136(5): 639–644. 10.4039/n03-099

[B6] Ledford J Paquin P Cokendolpher J Campbell J Griswold C (2011) Systematics of the spider genus *Neoleptoneta* Brignoli, 1972 (Araneae: Leptonetidae) with a discussion of the morphology and relationships for the North American Leptonetidae. Invertebrate Systematics 25: 334–388. 10.1071/IS11014

[B7] Oi R (1952) A new spider of the genus *Leptoneta*. Arachnological News 1: 10–12.

[B8] Seo BK (2016) Four new species of the genus *Falcileptoneta* (Araneae, Leptonetidae) from Korea. Journal of Species Research 5(3): 590–595. 10.12651/JSR.2016.5.3.590

[B9] Seo BK (2015) Ten new species of the genus *Falcileptoneta* (Araneae, Leptonetidae) from Korea. Korean Journal of Environmental Biology 33(3): 290–305. 10.11626/KJEB.2015.33.3.290

[B10] Wang CX Li SQ (2011) A further study on the species of the spider genus *Leptonetela* (Araneae: Leptonetidae). Zootaxa 2841: 1–90. 10.11646/zootaxa.2841.1.1

[B11] Wang CX Xu X Li SQ (2017) Integrative taxonomy of *Leptonetela* spiders (Araneae, Leptonetidae), with descriptions of 46 new species. Zoological Research 38(6): 321–448. 29280363 10.24272/j.issn.2095-8137.2017.076 PMC5767556

